# Impella‐Protected High‐Risk Percutaneous Coronary Intervention in the Elderly: Balancing Feasibility and Necessity

**DOI:** 10.1161/JAHA.125.042149

**Published:** 2025-05-06

**Authors:** Margriet Bogerd, José P.S. Henriques

**Affiliations:** ^1^ Department of Cardiology Amsterdam University Medical Center Amsterdam the Netherlands

**Keywords:** Editorials, elderly, feasibility, high‐risk percutaneous coronary intervention, impella‐protected, necessity, revascularization, Percutaneous Coronary Intervention, Revascularization

In this issue of the *Journal of the American Heart Association* (*JAHA*), Jakob et al present insightful data of patients included in the PROTECT III (Prospective, Multi‐Center, Randomized Controlled Trial of the IMPELLA RECOVER LP 2.5 System Versus Intra Aortic Balloon Pump in Patients Undergoing Non Emergent High Risk Percutaneous Coronary Intervention) cVAD registry, with the aim of assessing outcomes, including safety and efficacy, of older patients undergoing high‐risk percutaneous coronary intervention (HRPCI).^1^

The PROTECT III registry was a prospective, single‐arm, multicenter observational registry including patients with complex coronary artery disease (CAD) and severely depressed left ventricular systolic function, undergoing Impella‐supported HRPCI.^2^ The authors compared baseline characteristics and outcomes of 744 younger patients (younger than 75 years) with 493 older patients (older than 75 years; mean age, 82 years). Nearly half of the patients were admitted for angina and approximately two‐thirds for acute myocardial infarction (MI), of whom the majority (>80%) presented with non–ST‐segment–elevation MI (NSTEMI). The older cohort had significantly lower rates of diabetes and prior MI but slightly higher rates of hypertension and dyslipidemia, as well as clinically relevant worse kidney function. In addition, older patients had more severe valvular heart disease, yet a higher left ventricular ejection fraction on average. The baseline Synergy Between PCI With Taxus and Cardiac Surgery (SYNTAX) scores were high (>27) and comparable between the younger and older cohorts. Left main disease was more common in the older cohort (68% versus 52%, *P*<0.01). The treatment approach also differed, with older patients more often treated with a femoral approach and atherectomy used more frequently. The post‐PCI SYNTAX scores were slightly lower in the older population. Although presented in the supplemental material, it should be noted that periprocedural complications (including cardiac perforation, tamponade, cardiogenic shock, and life‐threatening, disabling, or major bleedings [BARC (Bleeding Academic Research Consortium) ≥3a]) were more prevalent in older patients, as was the prevalence of acute renal and respiratory dysfunction. The authors hypothesize that this may be associated with greater lesion complexity, more severe valvopathy, and a higher burden of noncardiac comorbidities in older patients. Vascular complication incidence rates were not significantly different between the groups. Importantly, all‐cause death and major adverse cardiac and cerebrovascular event rates were comparable between the groups at 30 and 90 days. However, age older than 75 years was a statistically significant predictor of all‐cause death at 1 year, even after adjustment for left ventricular ejection fraction, estimated glomerular filtration rate, pre‐PCI SYNTAX score, and left main PCI (adjusted hazard ratio, 1.99 [95% CI, 1.24–3.18], *P*=0.04). Overall, the authors conclude that Impella‐supported HRPCI in older patients is feasible, with comparable efficacy and an acceptable safety profile, despite more extensive and complex CAD.

The topic is highly relevant to date given the aging population and the increasing prevalence of (extensive) CAD, as the authors state in the introduction.^3^, ^4^ Hence, the question on how to treat elderly patients with extensive CAD thereby becomes increasingly prominent in daily clinical practice.^5^ However, before addressing the feasibility of Impella‐supported HRPCI in older patients, there are some prior questions that need to be addressed.^5^

## FIRST, SHOULD WE REVASCULARIZE OLDER PATIENTS WITH EXTENSIVE CAD?

In cases of acute coronary syndrome, specifically ST‐segment–elevation MI (STEMI), there is substantial evidence suggesting that older patients benefit more from complete revascularization compared with culprit‐only revascularization.^6^ A recent individual patient data meta‐analysis of 7 randomized controlled trials, including 1733 patients aged ≥75 years, showed that complete revascularization reduced the risk of death, MI, and ischemia‐driven revascularization up to 4 years.^6^ Noteworthy, the EXPLORE (Evaluating Xience and Left Ventricular Function in PCI on Occlusions After STEMI) trial demonstrated that revascularization of concurrent chronic total occlusions following STEMI did not improve prognosis, even up to 10 years, compared with optimal medical therapy, although symptom relief was observed.^7^ Similarly, a recent meta‐analysis of all randomized data on chronic total occlusion revascularization found no prognostic benefit but confirmed symptom relief.^8^ These randomized controlled trials were not specifically focused on elderly patients.

For NSTEMI, the additive value of revascularization in older patients is questionable. A large individual patient data meta‐analysis of 6 small randomized controlled trials including 1479 patients older than 70 years, showed that routine invasive treatment did not reduce the risk of the composite end point of all‐cause mortality and MI at 1 year compared with conservative management.^9^ Noteworthy, a significantly lower risk of repeat MI or urgent revascularization was observed.^9^ This finding was reiterated by the SENIOR‐RITA trial, a large randomized controlled trial comparing invasive treatment with a conservative strategy in 1518 patients with NSTEMI older than 75 years, which again showed that an invasive strategy with coronary angiography and revascularization did not result in a significantly lower risk of cardiovascular death or nonfatal MI during a median follow‐up of 4.1 years.^10^ Similar to the individual patient data meta‐analysis, nonfatal MI occurred slightly less often in the invasive strategy group.^9^, ^10^

Considering patients with chronic coronary disease, the additive value of revascularization is even more questionable. Does revascularization, for instance, improve the prognosis of patients with severe ischemic cardiomyopathy? The REVIVED‐BCIS2 (Revascularization for Ischemic Ventricular Dysfunction) trial showed that in patients with severe ischemic left ventricular systolic dysfunction, revascularization by PCI did not result in a lower incidence of death from any cause or hospitalization for heart failure as compared with optimal medical therapy.^11^ This finding was consistent among all prespecified subgroups, including older patients (older than 70 years), patients with left ventricular ejection fraction <29%, and patients with left main stem disease. There was an early benefit of PCI with respect to quality of life, but the between‐group difference diminished over time due to the progressive improvement in scores in the optimal medical therapy group. In addition, cardiac function appeared to improve in both groups during the course of follow‐up and this was unrelated to treatment group assignment.

In conclusion, except for STEMI patients, there is mounting evidence that invasive revascularization might not improve prognosis compared with optimal medical therapy in diverse subpopulations. While invasive revascularization was often associated with symptom relief and lower incidences of recurrent MI, it is important to note that most trials were not blinded and did not include sham procedures, which may have introduced a nocebo effect.

Nevertheless, when revascularization is deemed necessary and coronary artery bypass grafting is clinically unattractive despite complex multivessel disease, HRPCI may offer a solution, which leads to my second question.

## IS (IMPELLA‐) PROTECTED HRPCI SUPERIOR TO UNPROTECTED HRPCI?

Patients undergoing HRPCI are at risk of hypotension, decompensated heart failure, shock, and arrythmias that possibly lead to rapid hemodynamic deterioration or even death. In older patients, these risks may be even more outspoken. It thus seems intuitive to provide hemodynamic support during HRPCI, serving as a safety net in case of hemodynamic compromise. Importantly, BCIS‐1 (Balloon Pump Assisted Coronary Intervention Study) showed that routine intra‐aortic balloon placement did not reduce the incidence of major adverse cardiac and cerebrovascular events at hospital discharge in patients undergoing HRPCI.^12^ Two years later, PROTECT II (Prospective, Multi‐Center, Randomized Controlled Trial of the IMPELLA RECOVER LP 2.5 System Versus Intra Aortic Balloon Pump in Patients Undergoing Non Emergent High Risk Percutaneous Coronary Intervention), which randomized patients with HRPCI to either intra‐aortic balloon– or Impella 2.5–protected HRPCI, was published. As the authors rightfully mention, the PROTECT II trial was stopped prematurely for futility.^13^ Nevertheless, the Impella 2.5 provided better hemodynamic support and received Food and Drug Administration approval based on the per‐protocol analysis for the prespecified primary outcome analysis at 90 days. These studies led to a weak recommendation in the 2021 US guidelines for coronary artery revascularization, which states that in select high‐risk patients, elective insertion of an appropriate hemodynamic support device may be reasonable to prevent hemodynamic compromise during HRPCI.^14^

In their article, Jakob et al. present data from PROTECT III, a prospective registry that included 1134 “PROTECT‐II–like” patients treated with a more contemporary Impella device (2.5 or CP). The initial publication of PROTECT III compared major adverse cardiac and cerebrovascular event outcomes between patients from the PROTECT III registry and those from the PROTECT II trial and showed improved completeness of revascularization, less bleeding, and better 90‐day major adverse cardiac and cerebrovascular event outcomes in patients from the PROTECT III registry.^2^ While these findings might suggest that the newer‐generation Impella devices provide better clinical outcomes, they do not provide proof that Impella‐protected HRPCI is superior to unprotected HRPCI. In fact, a large single‐center cohort study from the MedStar Washington Hospital Center, which analyzed data from 1680 patients, demonstrated the feasibility and safety of unprotected HRPCI.^15^ The same research group performed an inverse propensity weighting analysis, comparing their institution's non–Impella‐protected HRPCI data with the published PROTECT III data, which showed a lower 90‐day mortality rate in the non‐Impella HRPCI group.^16^

Performing HRPCI under an Impella safety net seems intuitive and may facilitate more complete revascularization, which likely explains its increasing use.^2^, ^17^ However, Impella support comes at the cost of increased adverse events and device‐related costs.^17^ In order to mitigate vascular complications, in our center we routinely perform a preprocedural computed tomography scan of the whole arterial tree. However, to truly determine whether the benefits of Impella protection in HRPCI outweigh its risks and costs, randomized data are essential. We eagerly await the results of the PROTECT IV (Impella‐Supported PCI in High‐Risk Patients With Complex Coronary Artery Disease and Reduced Left Ventricular Function) trial (NCT04763200), which will compare Impella‐supported with nonsupported HRPCI in patients up to 90 years old.

Until then, the decision to proceed with invasive revascularization, and whether to use Impella protection, should be carefully weighed on an individual basis. This evaluation should take into account the patient's clinical profile and anatomy, as well as the patient's and physician's preferences (Figure). As the landscape of revascularization is evolving, there may be cases where refraining from invasive revascularization is the better option.

**Figure 1:**
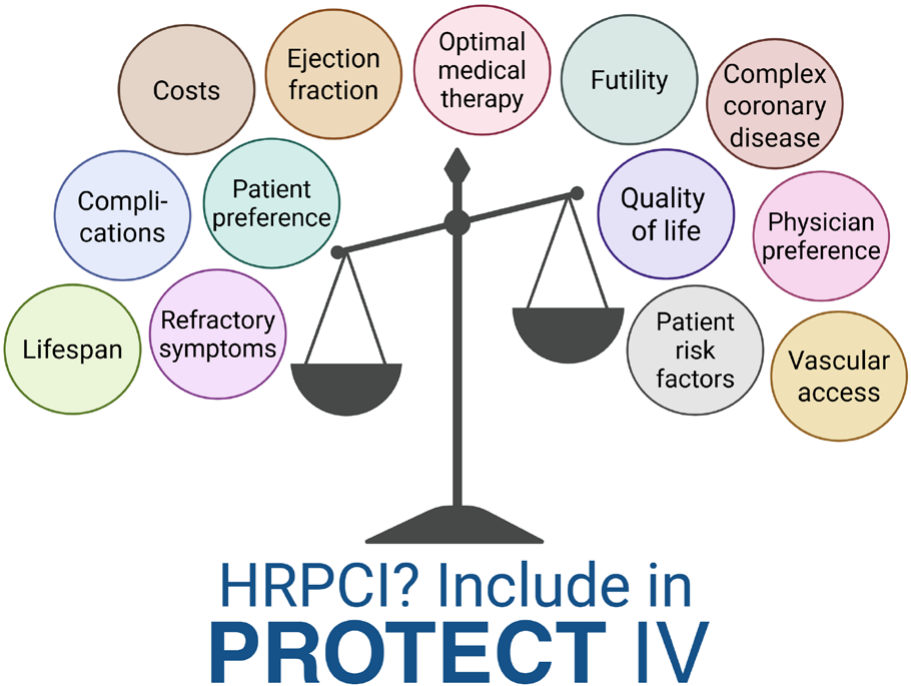
Protected HRPCI in the elderly: balancing feasibility and necessity. HRPCI indicates high‐risk percutaneous coronary intervention. Created in BioRender. Henriques J. 2025 https://BioRender.com/nfe8u71.

The present study demonstrated that Impella‐protected HRPCI is a feasible option in the elderly, with comparable efficacy as in younger patients and with an acceptable safety profile. However, older patients were at increased risk for periprocedural complications and an age of 75 years or above was a statistically significant predictor of 1‐year all‐cause mortality.

Since the benefit of protected versus unprotected HRPCI has not yet been established in a randomized trial, we recommend that all patients with HRPCI be considered for inclusion in the RECOVER IV trial. Finally, we underscore the authors' remark that older patients must be informed of the additional risks associated with Impella‐supported HRPCI to ensure well‐informed and shared decision‐making.

## Disclosures

None.
